# How to approach and treat VAP in ICU patients

**DOI:** 10.1186/1471-2334-14-211

**Published:** 2014-04-30

**Authors:** Bárbara Borgatta, Jordi Rello

**Affiliations:** 1 Critical Care Department, Hospital Universitario Vall d’Hebron, Barcelona, Spain; 2 Vall d’Hebron Institute of Research, Barcelona, Spain; 3 CIBERES, Barcelona, Spain; 4 Universitat Autònoma de Barcelona, Barcelona, Spain

**Keywords:** Ventilator-associated pneumonia, Nosocomial pneumonia, Treatment, Antibiotic, Management

## Abstract

**Background:**

Ventilator-associated pneumonia (VAP) is one of the most frequent clinical problems in ICU with an elevated morbidity and costs associated with it, in addition to prolonged MV, ICU-length of stay (LOS) and hospital-length of stay. Current challenges in VAP management include the absence of a diagnostic gold standard; the lack of evidence regarding contamination *vs.* airway colonization *vs*. infection; and the increasing antibiotic resistance. We performed a Pubmed search of articles addressing the management of ventilator-associated pneumonia (VAP). Immunocompromised patients, children and VAP due to multi-drug resistant pathogens were excluded from the analysis. When facing a patient with VAP, it’s important to address a few key questions for the patient’s optimal management: when should antibiotics be started?; what microorganisms should be covered?; is there risk for multirresistant microorganisms?; how to choose the initial agent?; how microbiological tests determine antibiotic changes?; and lastly, which dose and for how long?. It’s important not to delay adequate treatment, since outcomes improve when empirical treatment is early and effective. We recommend short course of broad-spectrum antibiotics, followed by de-escalation when susceptibilities are available. Individualization of treatment is the key to optimal management*.*

## Background

Ventilator-associated pneumonia (VAP) is one of the most frequent clinical problems in ICU. With an estimated incidence from 5–20 cases per 1.000 mechanical ventilation (MV) days; which has decreased over the last decade with the implementation of care bundles. However it still remains as the most frequent infection amongst critically ill patients and as the main cause of antibiotic prescription in ICU [1-4]. Despite presenting a low attributable mortality (less than 10%); its burden relies on the elevated morbidity and costs associated with it, such as an estimated excess of cost as high as $40,000 per patient’s episode, in addition to prolonged MV, ICU-length of stay (LOS) and hospital-length of stay [2,5,6].

VAP represents 80% of hospital-acquired pneumonia (HAP) and is defined as pneumonia developing after 48-72 h of MV. Many screening and diagnostic criteria have been used in order to an early identification of VAP and differentiation from ventilator-associated tracheobronquitis (VAT), with suboptimal results since radiological findings in the critically ill lack sufficient sensitivity and specificity. Recently, Centers for Disease Control and Prevention (CDC) and Klompas *et al*. have established a new surveillance strategy for the screening of infection-related ventilator-associated complications (IVAC) [7] that represents a major change in VAP diagnosis paradigm, and focusing on sustained hypoxemia (lasting 2 calendar days) as a *sine qua non* characteristic of VAP, even in the absence of clear findings in the Rx. IVAC include all patients with 3 or more days of MV; with worsening of oxygenation lasting 2 calendar days, identified as increase in FiO2 or PEEP; which can be classified as *possible VAP* and *probable VAP*, depending on the criteria they meet.

Current challenges in VAP management include the absence of a diagnostic gold standard; the lack of evidence regarding contamination *vs.* airway colonization *vs*. infection; and the increasing antibiotic resistance.

## VAP management

**Figure 1:**
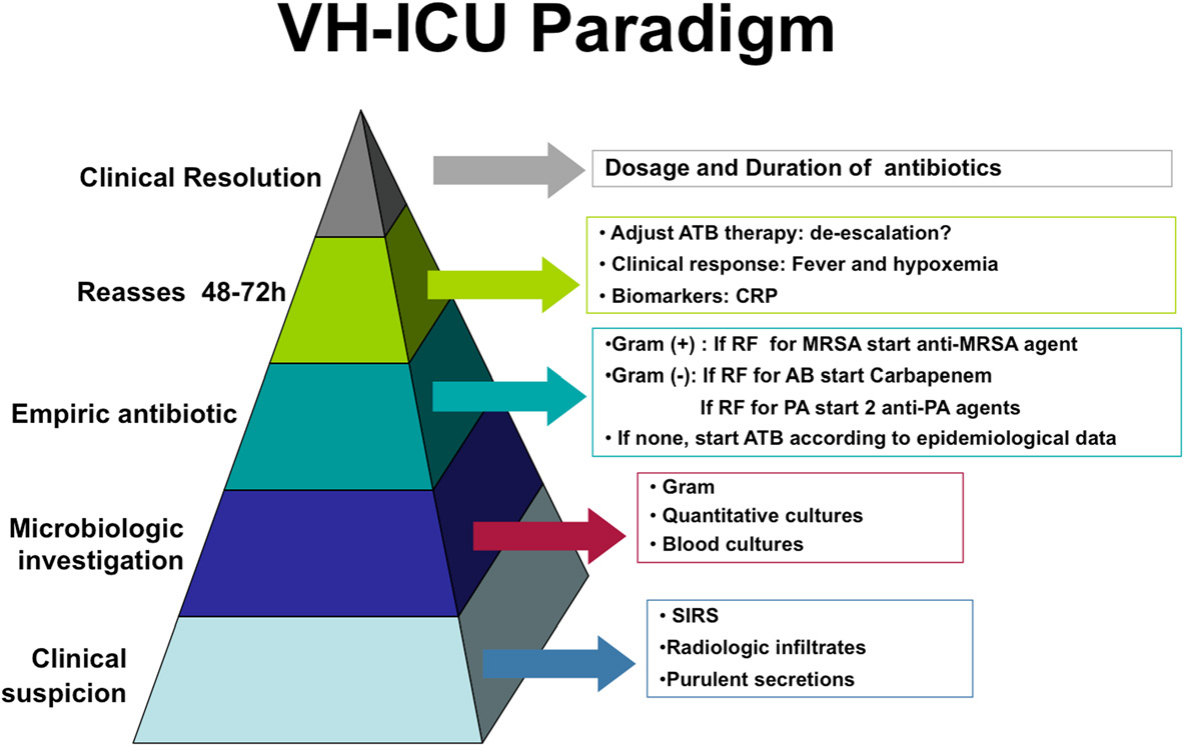
**VH-ICU Paradigm for VAP.** AB: *Acinetobacter baumanii*, ATB: antibiotic, CRP: C-reactive protein, MRSA: methicilin-resistant *Staphilococcus aureus*, PA: *Pseudomonas aeruginosa*, SIRS: systemic inflammatory response syndrome.

When facing a patient with VAP, it’s important to address a few key questions for the patient’s optimal management: when should antibiotics be started?; what microorganisms should be covered?; is there risk for multirresistant microorganisms?; how to choose the initial agent?; how microbiological tests determine antibiotic changes?; and lastly, which dose and for how long? See Figure 1*.*

### Antibiotic start and choice

It has been well documented that delayed effective therapy increases morbidity and mortality rate among patients with VAP [3,8]. Indeed, changing to an active agent after microbiology reports may not improve patient’s outcomes [9]. Initial antibiotic should be active against likely pathogens; therefore its choice should be based on prior antibiotic exposure, patient co-morbidities, length of hospitalization and local epidemiology. Special considerations should be taken with patients with Health-care associated pneumonia (HCAP), since causative organism differs with higher probability for multi-drug resistant (MDR) pathogens. This subset of patients include recent hospitalization in acute care facility (<90 days), resides in a nursing home or long-term care facility; received recent intravenous antibiotic therapy, chemotherapy, or wound care within the past 30 days of the current infection; or attended a hospital or hemodialysis clinic [10].

We support prompt antibiotic initiation with a short course of broad spectrum antibiotics, followed by de-escalation when susceptibilities are available [11]; stressing that initial narrow-spectrum antibiotic should not be used. Certainly, besides microbiological sensitivities, lung penetration of active agents is a crucial matter that has to be considered.

Combined therapy is a long established practice in ICU, especially in VAP caused by *P. aeruginosa* because of its high rates of resistance and initial ineffective antibiotic therapy [12]. Many studies support that in bacteremic infections and VAP due to *P. aeruginosa,* combination therapy improves appropriate empirical therapy [10,13-15]; moreover a meta-analysis was able to detect reduced mortality in this subset of patients (OR 0.50, 95% CI 0.30-0.79), and not in infection due to other gram-negative bacilli [13]. When analyzed by severity-of-illness, combined therapy in patients at high risk of death is significantly associated with reduced mortality only in the subset of patients with shock, whereas patients without shock have worse outcomes, probably due to toxicity [16,17].

### Predicting causative organism

Overall, VAP’s main causative microorganisms are *Pseudomonas aeruginosa* and *Staphylococcus aureus*[3]. When considered by the time of onset, early-VAP (within the first 4 days of MV) is usually associated with normal oropharynx flora; such as *S. pneumoniae, S. aureus and H. influenzae.* However, a multicenter study showed a high prevalence (50.7%) of potentially resistant microorganisms (PMR) in this subset of patients with no risk factors for PMR [18]. Late-VAP is largely caused by aerobic Gram-negative bacilli, of which up to 70% of cases are due to *P. aeruginosa*, *Acinetobacter baumannii*, or methicilin-resistant *S. aureus* (MRSA). Also differences are seen in surgical and neurological patients, where *S. aureus* is the main pathogen [1-3].

Strategies based on guidelines are accurate for predicting causative microorganism and thus appropriate initial antibiotic in VAP (97.9%, p < 0.05), but also are endotracheal aspirates from samples retrieved 2 days before the onset of VAP [19].

### Tailoring antibiotic treatment

Standard antibiotic dosage has proven to be insufficient (under-dosage) in critically ill patients with severe sepsis, especially when they undergo continuous renal replacement or ECMO therapies [20,21]. A recent multicenter study addressing antibiotic levels in ICU patients treated with standard doses of betalactams, showed that 16% of them do not meet adequate levels and that it was associated with worse outcomes, while patients who achieved 50% and 100% ratios of free antibiotic concentrations above the minimum inhibitory concentration of the pathogen were associated with positive clinical outcome (OR: 1.02 and 1.56, respectively, p < 0.03) [22]. Suggesting that antibiotic dosage and form of administrations should be personalized in order to improve patient’s outcomes.

A new approach in VAP treatment is the use of nebulized antibiotics. Its main appeal is that allows achieving high local concentration of antibiotics, with fast clearance, which reduces risk for development of resistance, and with minimal absorption that translates into less toxicity. Even though many issues have to be improved, such as effective delivery systems and optimal formulations that are able to reach the alveoli and are well tolerated by the patients [23]; it poses as a desirable strategy for VAP antibiotic treatment, especially in multirresistant strains where active agents have elevated risk of toxicity. Disadvantages include frequent ventilator’s filter obstruction, which some groups solve by routinely change after each administration [24].

Recent studies are lacking of robust data, in spite of which, nebulized therapy has shown to be effective. Nebulized monotherapy has proven to be non-inferior to IV therapy; and as adjunctive to IV regimens is associated with higher antibiotic concentrations at target tissue and less number of antibiotics per patient per day [24-28], and in some cases respiratory eradication of the microorganism [24,29]. Available formulations for nebulization include tobramycin, aztreonam, ceftazdime, amikacyn and colistin.

### Duration of treatment

Optimal duration of antibiotic therapy is still controversial. Until recently, it was standard practice antibiotic regimens of minimum 15 days for uncomplicated infections [3]. Current trends favor short courses of antibiotics of 7–8 days if patient’s response is satisfying; always individualizing to resolution. This approach is has equivalent clinical cure rates than long courses [30] and enables the reduction of side effects, costs and development of resistant phenotypes [3]. A recent meta-analysis concluded that short courses are associated with more antibiotic free days without any detrimental effect on mortality, besides the fact that prolonged antibiotic courses do not prevent recurrences [30,31]. Not to mention that in patients with VAP and negative bronchoalveolar lavage cultures, early antibiotic discontinuation does not affect mortality and is associated with fewer respiratory and multidrug resistant superinfections (10.0% vs. 28.6% and 7.5% vs. 35.7%, p < 0.05 respectively) [32].

### Optimization of antibiotics

Optimization of antibiotics does not mean strictly following guidelines; instead it means empowerment, stewardship and team working. Antibiotic stewardship is a simple and cost-effective way to improve clinical outcomes while minimizing antibiotic side effects and its negative consequences; maintaining quality of care [33,34].

## What’s next?

Research should be directed towards the development of ultra-fast diagnostic techniques that can immediately predict causative microorganism, without the need of specimen processing and also detect multirresistance mechanisms to avoid inadequate initial antibiotic treatment.

## Conclusions

*Getting it right the first time:* It’s important not to delay adequate treatment, since outcomes improve when empirical treatment is early and effective. *Prompt appropriate therapy, then step-down:* we recommend short course of broad-spectrum antibiotics, followed by de-escalation when susceptibilities are available. *Individualize always!:* regarding dosage, way of administration and duration based on clinical response.

## Abbreviations

CDC: Centers for disease control and prevention; HAP: Hospital-acquired pneumonia; HCAP: Health-care associated pneumonia; LOS: Length of stay; MDR: Multi-drug resistant; MRSA: Methicilin-resistant *S. aureus*; MV: Mechanical ventilation; PMR: Potentially resistant microorganisms; VAP: Ventilator-associated pneumonia; VAT: Ventilator-associated tracheobronquitis.

## Competing interest

Jordi Rello has served in the as board and speaker bureaus for Pfizer, Astellas, and Cubicin. Bárbara Borgatta has no competing interest.

## Authors’ contribution

BB and JR contributed equally to the writing of this manuscript. All authors read and approved the final manuscript.

## Pre-publication history

The pre-publication history for this paper can be accessed here:

http://www.biomedcentral.com/1471-2334/14/211/prepub
